# An integrated community‐based outpatient therapeutic feeding programme for severe acute malnutrition in rural Southern Ethiopia: Recovery, fatality, and nutritional status after discharge

**DOI:** 10.1111/mcn.12519

**Published:** 2017-10-10

**Authors:** Elazar Tadesse, Amare Worku, Yemane Berhane, Eva‐Charlotte Ekström

**Affiliations:** ^1^ Department of Women's and Children's Health Uppsala University Uppsala Sweden; ^2^ Addis Continental Institute of Public Health Addis Ababa Ethiopia; ^3^ Kotebe Metropolitan University Addis Ababa Ethiopia

**Keywords:** Ethiopia, integrated outpatient therapeutic programme, outcome, recovery, severe acute child malnutrition

## Abstract

A scaled up and integrated outpatient therapeutic feeding programme (OTP) brings the treatment of severely malnourished children closer to the community. This study assessed recovery from severe acute malnutrition (SAM), fatality, and acute malnutrition up to 14 weeks after admission to a programme integrated in the primary health care system. In this cohort study, 1,048 children admitted to 94 OTPs in Southern Ethiopia were followed for 14 weeks. Independent anthropometric measurements and information on treatment outcome were collected at four home visits. Only 32.7% (248/759) of children with SAM on admission fulfilled the programme recovery criteria at the time of discharge (i.e., gained 15% in weight, or oedema, if present at admission, was resolved at discharge). Of all children admitted to the programme for whom nutritional assessment was done 14 weeks later, 34.6% (321/928) were severely malnourished, and 37.5% (348/928) were moderately malnourished; thus, 72.1% were acutely malnourished. Of the children, 27/982 (2.7%) had died by 14 weeks, of whom all but one had SAM on admission. Children with severe oedema on admission had the highest fatality rate (12.0%, 9/75). The median length of admission to the programme was 6.6 weeks (interquartile range: 5.3, 8.4 weeks). Despite children participating for the recommended duration of the programme, many children with SAM were discharged still acutely malnourished and without reaching programme criteria for recovery. For better outcome of OTP, constraints in service provision by the health system as well as challenges of service utilization by the beneficiaries should be identified and addressed.

## 1 INTRODUCTION

In low‐income settings, severe acute malnutrition (SAM) is common among children below 5 years of age and associated with high rates of child morbidity and mortality (WHO, 2013; WHO & UNICEF, 2009). Previously, management of SAM was limited to inpatient care in health facilities and therapeutic feeding centres (UNICEF, 1990), but currently, community‐based management of acute malnutrition is recommended as the standard management for all SAM cases (WHO, WFP, & UNICEF, 2007; WHO, 2013). Community‐based management of acute malnutrition has both a community‐based outpatient therapeutic feeding programme (OTP) for uncomplicated cases and a facility‐based inpatient care for complicated cases. It also includes treatment of moderate acute malnutrition and community mobilization activities (Ashworth, 2006; Gatchell, Forsythe, & Thomas, 2006; WHO et al., 2007). The shift to managing uncomplicated SAM through OTPs was made possible through the development of ready‐to‐use therapeutic food (RUTF) that provides all the energy and nutrients required for rehabilitation of children with SAM and can be safely fed to children with SAM at their homes (Kapil, 2009; Nutriset, 2010). Moreover, simplified guidelines for identifying and managing SAM were developed to support the community health workers responsible for these activities in the community (Chamois, 2009; FMOH, 2007).

Most of the OTPs were initially small‐scale and implemented by externally funded non‐governmental organizations as part of emergency relief programmes (Chaiken, Deconinck, & Degefie, 2006; Collins et al., 2006; Collins & Sadler, 2002; Manary & Sandige, 2008). Although there are reports of good recovery rates from such OTPs (Ashworth, 2006; Chaiken et al., 2006; Chamois, 2009; Collins et al., 2006; Collins & Sadler, 2002), programmes integrated to government health system at the beginning of scaling and integration of OTP had recovery lower than the small‐scale programmes (Hedwig, Swindale, Grant, & Navarro‐Colorado, 2008). This reflects the knowledge that the context into which nutrition interventions are integrated is modifying their effect (Victora et al., 2005). Reports on the performance of programme after the scaling up and integration into government health systems are scarce. Further, the focus of studies has commonly been the children's health and nutritional status at discharge, usually 6–8 weeks after admission, and little is known to what extent the benefits of the programme are sustained beyond discharge. A concern is also that most of these reports are based on data excerpted from programme records and not by independent assessments (Chaiken et al., 2006; Collins, 2001; Collins & Sadler, 2002). Set in Southern Ethiopia in an area of government‐implemented OTP, the aim of our study was to use independently collected data to assess recovery, fatality, and acute malnutrition on and beyond discharge from the programme.

**Key messages**

- Children with severe oedema at admission who should have been referred to inpatient care had a high case fatality rate.
- The vast majority of children with severe acute malnutrition on admission failed to reach programme recovery criteria despite programme stay of recommended duration.
- Many of those with severe acute malnutrition on admission remained severely malnourished after 14 weeks of follow‐up.

## 2 METHODS

### 2.1 Study design and setting

This cohort study was conducted from July to December 2011 and included children admitted to OTPs in 94 health posts in four adjacent districts of Wolaita Zone in Southern Ethiopia. With a mostly rural population (88%), the area is characterized by a high population density (342/km^2^), a fragmentation of farmland ownership and limited income‐generating opportunities (Hailu & Regassa, 2007; Teklu, 2003). Moreover, recurrent drought and subsequent crop failure in the area have resulted in cyclical nutritional emergencies and chronic food insecurity (Hailu & Regassa, 2007; The Socioeconomic Profile of Southern Nations, Nationalities and Peoples Region, 2000). Food insecurity gets worse during preharvest months (May–July) and starts to improve during harvest (August–November) and postharvest (December–February) time. Thus, our study period covers a range of food insecurity exposures.

From 2008, the Ethiopian government has scaled up and integrated the OTPs into the existing public health system, including the health posts (Chamois, 2009). Health posts are the lowest tier in the Ethiopian health system hierarchy, serving approximately 5,000 people and being staffed by two female health extension workers, who provide basic curative and preventive health services (Wakabi, 2008). Although the World Health Organization (WHO) has recommended a cut‐off at mid‐upper arm circumference (MUAC) < 115 mm (WHO, 2013) to define SAM, the Ethiopian national SAM management protocol uses a MUAC cut‐off <110 mm or oedema to admit children into the OTP (FMOH, 2007). After admission, the children's caregivers are to be provided with RUTF, medication, and counselling, after which they return home to manage the child with SAM on their own (FMOH, 2007).Visits to the health post for check‐ups and refilling of RUTF are usually scheduled weekly.

### 2.2 Enrolment and follow‐up procedures

A weekly visit to all the health posts in the selected districts was undertaken to identify children participating in the OTP. Children aged from 6 to 59 months, from whom the research team collected anthropometric data within 7 days of admission, were eligible to participate in the study. Data were collected at four households visits, the first within 1 week of admission and then after 4, 8, and 14 weeks. The follow‐up at 4 and 8 weeks was selected to capture anthropometric change while in OTP and at around expected time of discharge (termination of treatment) as programme allowed a maximum participation of 8 weeks. The follow‐up at 14 weeks was selected arbitrarily to examine acute malnutrition after allowing a reasonable time after expected time of discharge for some change in nutritional status to have occurred.

### 2.3 Data collection and instruments

Children's MUAC was measured using WHO‐recommended MUAC tape and techniques, and the presence of bilateral pitting oedema was assessed using the recommended techniques (WHO & UNICEF, 2009). Weight (to the nearest 0.1 kg) was measured using UNICEF‐recommended digital electronic scale. A structured questionnaire that included questions about socio‐demographic information on household, caregiver, and children was used to collect data. Twenty‐three female nurses collected data and were trained in anthropometric measurement techniques including repeated standardization sessions to ensure accuracy and precision of measurements according to established guidelines (FMOH, 2007).

### 2.4 Outcome measures and statistical analysis

The presence of acute malnutrition was defined by use of MUAC or presence of oedema according to WHO and national recommendations (FMOH, 2007; WHO et al., 2007). Children with oedema on admission were further categorized into two groups based on severity: mild/moderate oedema and severe oedema. Children without oedema were categorized into three groups: (a) most severely wasted (MUAC < 110 mm); (b) less severely wasted (MUAC 110–114 mm); and (c) not severely wasted (MUAC ≥ 115 mm). Nutritional status at discharge and 14 weeks after admission was categorized in three groups of acute malnutrition as defined by WHO (WHO, 2013).

For children with SAM at admission programme outcome was analysed, and the evaluation criteria included assessment of parameters used in programme both for individual decisions regarding discharge (recovery and length of admission) and for assessing programme performance in line with Sphere standard (Sphere Project, 2011; i.e., case fatality, proportion of recovery, average length of stay in programme, proportion of defaulters, and average weight gain; FMOH, 2007; Sphere Project, 2011). According to Sphere, the definition of recovery from SAM is based on SAM management guidelines stipulating gain of 15% of weight at admission for nonoedematous children and resolution of oedema for children with oedema in order to discharge children (FMOH, 2007; WHO & UNICEF, 2009). Average weight gain is a programme indicator that according to the national guideline is calculated as weight gained (g) per body weight at admission (kg) per day for children who recovered (FMOH, 2007). We also calculated average weight gain among all children admitted with SAM regardless whether they had recovered or not. The average weight gain indicator was not calculated for children with oedema at admission. Defaulters from OTP were children who missed health post follow‐up visits for at least 2 weeks.

Data were entered into Epi‐Info (Version 6.0), cleaned, and exported to SPSS for Windows (Version 20. 0) for analysis. Children with missing key variables (MUAC or oedema status on admission, age, and sex) were excluded from analysis. Data were explored for levels of missing values, and almost all factors had missing value less than 5%. Length of programme stay and weight gain were not normally distributed when normality test (Shapiro–Wilk) was applied; thus, log transformed and normality were obtained for both variables. Nutritional status on admission, discharge, and 14 weeks after initial admission were presented as proportions of children with 95% confidence intervals (CI) for categorical outcomes.

### 2.5 Ethical considerations

The research protocol was reviewed and approved by the regional health bureau of South Ethiopia, district health bureaus, the Ethical Review Board at Addis Continental Institute of Public Health, Addis Ababa, and Uppsala Regional Ethical Review Board. The Helsinki Declaration was applied in the research project, including gaining of informed consent.

## 3 RESULTS

**Figure 1:**
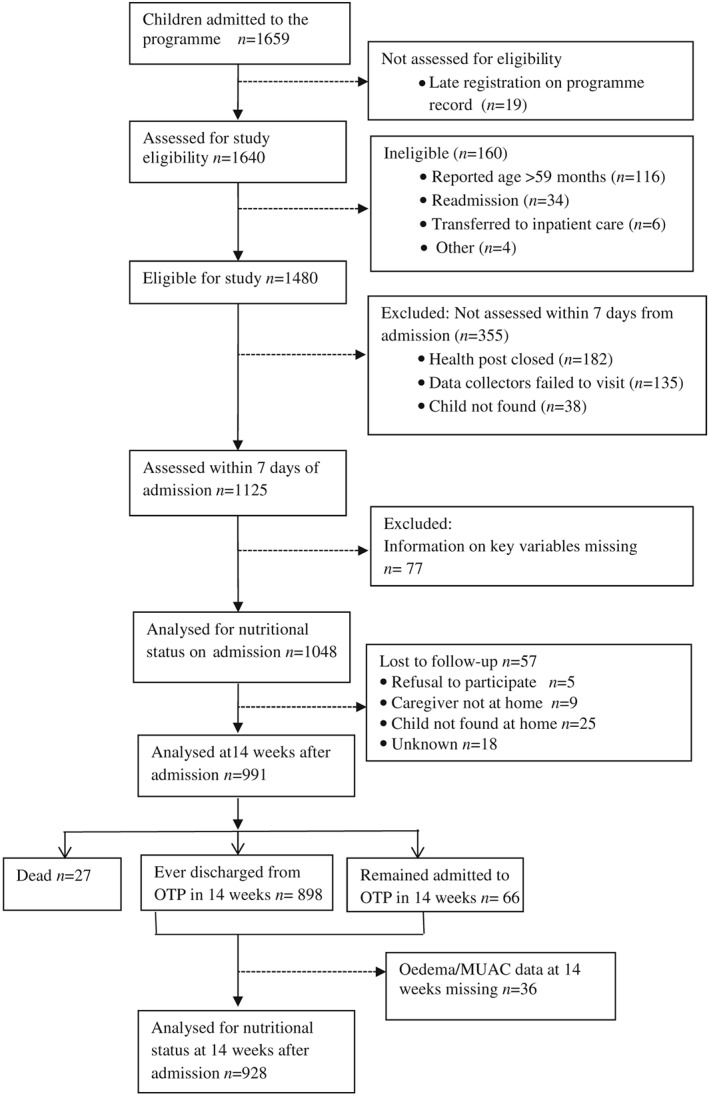
Flow of children admitted to the outpatient therapeutic programme (OTP). MUAC = mid‐upper arm circumference

As shown in Figure 1, children with acute malnutrition were followed from admission in a community‐based therapeutic programme up to 14 weeks. Of 1,480 eligible children (6–59 months of age), 355 were excluded from the study because anthropometric assessments were not done within 7 days of admission, and the provided nutritional therapy might have changed the baseline nutritional status. The main reasons for these losses were that health posts were closed so data collectors could not get access to OTP records to look for new admissions (*n* = 182) and that the data collectors failed to visit mainly due to time constraints in the field (*n* = 135). Further, of 1,125 children included in the study, nutritional status on admission was analysed for 1,048 children due to missing of key variables (*n* = 77). During the 14 weeks of the study, 57 children were lost to follow‐up mainly as they were not found (*n* = 25), and 27 children had died. The final analysis was performed on 928 children for whom the nutritional status was measured at follow‐up 14 weeks after the initial admission. The relative large number of children excluded from analyses was compared with children included, and no important differences in terms of baseline characteristics as child age, sex, or caregivers' and household characteristics were found.

### 3.1 Demographic and socio‐economic characteristics of households and caregivers

Most of the participants lived in houses with thatched roof and wood and mud or grass walls (74.7%), had an open‐pit latrine (75.5%), and collected water from a public tap or a protected well or spring (77.0%). At the time of the study, 42.4% of the households had more than one child less than 5 years of age. The caregivers of children admitted to the OTP were mostly biological mothers, married, and on average, they were 30.6 years old. The majority of children were below 24 months of age (62.3%), and the female‐to‐male ratio was 1.3 (Table 1).

**Table 1:** Characteristics of the children admitted to outpatient therapeutic programme and their caregivers

Child characteristics *N* = 1,048	*n*/*N*	%
Sex		
Boys	458/1,048	43.7
Girls	590/1,048	56.3
Age (in months)		
6–11	366/1,048	34.9
12–23	287/1,048	27.4
24–35	126/1,048	12.0
36–47	162/1,048	15.5
48–59	107/1,048	10.2
Caregiver characteristics *N* = 1,021		
Relationship to child		
Biological mother	903/1,019	88.6
Marital status		
Married	893/992	90.0
Age (in years)		
15–19	15/1,006	1.5
20–29	409/1,006	40.7
30–39	480/1,006	47.7
≥40	117/1,006	11.6
Current occupation		
No job	150/1,020	14.7
Farmer	542/1,020	53.1
Petty trade	328/1,020	32.2
Educational status		
Never attended school	34/1,020	3.3
In but did not complete primary school	708/1,020	69.4
Completed primary/secondary school	278/1,020	27.3
Household characteristics *N* = 1,021		
Sanitation		
Pit latrine with slab	27/1,019	2.6
Open pit	769/1,019	75.5
Open space (bush/farm land, other)	223/1,019	21.9
Source of drinking water		
Protected source (public tap/protected well/spring)	783/1,017	77.0
Unprotected source (spring/wall/other)	234/1,017	23.0
House construction		
Corrugated iron roof with wood and mud wall	258/1,019	25.3
Thatch roof with wood and mud/grass wall	761/1,019	74.7
Number of under five children		
One	588/1,021	57.6
More than one	433/1,021	42.4

### 3.2 Nutritional status of children on admission to OTP

When using either the oedema or MUAC criteria to define SAM, 78.8% (826/1,048) of the admitted children had SAM on admission, and thus, 21.2% (222/1,048) did not have SAM. Of the admitted children, 20.5% (215/1,048) had SAM defined by oedema, with one out of three children having severe oedema. Among those without oedema at admission, 53.9% (449/833) had MUAC < 110 mm, and 19.4% (162/833) had MUAC 110–114 mm. The proportion of children with SAM was significantly higher among children less than 24 months of age (56.5%; 95% CI [52.7, 60.3] vs. 20.2; 95% CI [16.5, 24.4]) among the older children. In contrast, the proportion of children with oedema was significantly higher in children ≥24 months of age (18.2; 95% CI [14.6, 22.3,] vs. 0.8; 95% CI [0.3, 1.7]).

### 3.3 Nutritional status on discharge and the programme outcome

The nutritional status after discharge was analysed for 98.1% (881/898) of the children who were discharged within the 14 weeks of follow‐up. More than one‐third of discharged children (35.8%, 315/881) were severely malnourished, and 37.7% (330/881) were moderately malnourished; that is, 73.5% (645/881) were acutely malnourished on discharge (Table 2). There was no statistical significant change in proportion of SAM due to duration (days) between discharge and time assessment anthropometric assessment; that is, 39.0% (73/187; 95% CI [32.2, 46.2] ≤ 2 days post discharge) and 36.4% (52/116; 95% CI [28.8, 44.5] ≥ 28 days post discharge).

**Table 2:** Programme status and fatality of children at 14‐weeks of follow‐up, by nutritional status at admission to outpatient therapeutic programme

Nutritional status at admission *N* = 991	Still admitted		Discharged but readmitted		Discharged		Fatality rate	
	*n* (%)	95% CI	*n* (%)	95% CI	*n* (%)	95% CI	*n* (%)	95% CI
All children (*N* = 991)	66 (6.7)	5.2, 8.3	107 (10.8)	9.0, 12.9	791 (79.8)	77.2, 82.2	27 (2.7)	1.8, 3.9
All SAM (*n* = 787)	51 (6.5)	4.9, 8.4	99 (12.6)	10.4, 15.0	611 (77.1)	74.6, 80.5	26 (3.3)	2.2, 4.8
Severe oedema (*n* = 75)	0 (0.0)	0.0, 3.9	7 (9.3)	4.2, 17.6	59 (78.7)	68.3, 86.8	9 (12.0)	6.0, 20.9
Mild/moderate oedema (*n* = 136)	8 (5.9)	2.8, 10.9	16 (11.8)	7.1, 18.0	108 (79.4)	72.0, 85.6	4 (2.9)	0.9, 6.9
MUAC < 110 mm (*n* = 420)	35 (8.3)	6.0, 11.3	65 (15.5)	12.3, 19.2	309 (73.6)	69.2, 77.6	11 (2.6)	1.4, 4.5
MUAC 110–114 mm (*n* = 156)	8 (5.1)	2.4, 9.5	11 (7.1)	3.8, 11.9	135 (86.5)	80.5, 91.2	2 (1.3)	0.2, 4.2
All non‐SAM (*n* = 204)	15 (7.4)	4.3, 11.6	8 (3.9)	1.8, 7.3	180 (88.2)	83.3, 92.1	1 (0.5)	0.0, 2.4

*Note*. MUAC = mid‐upper arm circumference; SAM = severe acute malnutrition.

The proportion of children with SAM after discharge was significantly higher for those who had the most severe degree of wasting (MUAC < 110 mm) on admission to the programme (Table 2). When compared to other forms of SAM, children with most severe wasting (MUAC < 110 mm) on admission had highest proportion of SAM on discharge (52.9%; 95% CI [47.7, 58.0]). Moreover, 13.0% (24/184) of the children who were not severely malnourished on admission had become severely malnourished by the time of discharge from the programme.

**Figure 2:**
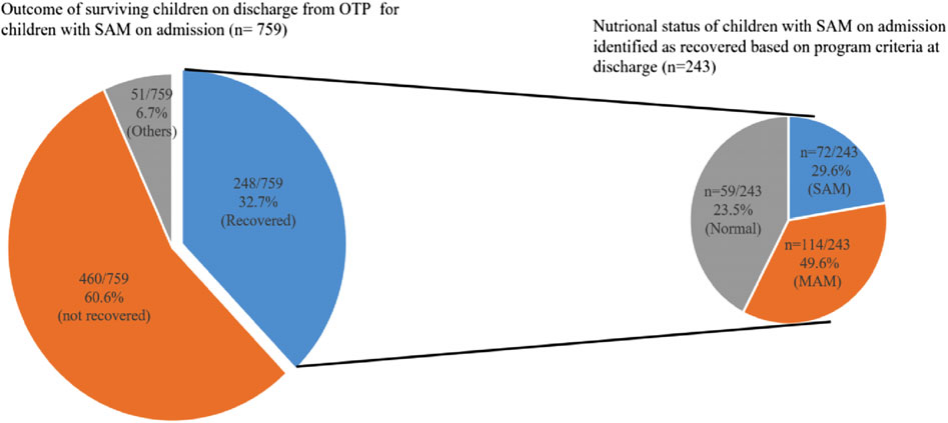
Nutritional status of children with severe acute malnutrition (SAM) on admission and exited the programme as “recovered” based on programme criteria. Recovered = gained 15% of admission weight or oedema resolved on admission. Non‐recovered = not gained 15% of admission weight or oedema not resolved. Other = transferred to inpatient care (*n* = 4), default (*n* = 18), and death (*n* = 17). SAM = MUAC < 115 mm and/or oedema. MAM (moderate acute malnutrition) = MUAC 115–124 mm and no oedema. Normal = MUAC ≥ 125 mm and no oedema. MUAC = mid‐upper arm circumference

For children who had SAM at admission, programme outcomes, that is, proportion of recovery, the average length of stay in the programme, the average weight gain, and proportion defaulters were computed. Of the 826 with SAM on admission, 91.9% (759/826) were included in the analysis of programme outcome. The remaining 8.1% (67/826) were not included because they were still in the programme (5.6%, 46/826) and 2.5% (21/826) had missing information. When all children admitted with SAM were considered, the mean length of stay in the programme was 6.8 weeks (95% CI [6.5, 7.0]). The median length of admission to the programme was 6.6 weeks [interquartile range: 5.3, 8.4 weeks]. Very few (4%) of the SAM children failed to participate and become defaulters in the programme. Recovery, that is, 15% weight gain or resolution of oedema, when applicable, at discharge, was low (32.7%, 248/759). The average weight gain among all nonoedematous SAM children was 2.5 g kg^−1^ day^−1^ (95% CI [2.2, 2.8]) and 4.9 g kg^−1^ day^−1^ (95% CI [4.4, 5.4]) if computed only for recovered nonoedematous SAM children according to the Sphere recommendation (Sphere Project, 2011). When WHO definition of acute malnutrition was applied, of the children who reached the 15% weight gain programme recovery criteria at discharge, 29.6% (72/243) were still severely malnourished (MUAC < 115 or oedema), and 46.9% (114/243) were moderately malnourished (MUAC 115–124 mm); thus, 76.5% (186/243) were acutely malnourished (Figure 2).

### 3.4 Programme status, fatality, and nutritional status at 14 weeks after admission to the outpatient therapeutic programme

Fourteen weeks after admission to the OTP, 90.6% (898/991) of all children had been discharged from the programme, but 10.8% (107/991) were readmitted (Table 2). Proportion of children readmitted was significantly higher among children with most severe degree of wasting on admission (MUAC < 110 mm) when compared to less severely degree of wasting (MUAC 110–114 mm) and those who were not severely malnourished (MUAC > 115 and no oedema) on admission; 15.5% (95% CI [12.3, 19.2]) versus 7.1% (95% CI [3.8, 11.9]) versus 3.9% (95% CI [1.8, 7.3]). Twenty‐seven children had died by 14 weeks, of whom all but one had SAM on admission. Nine of the deaths had occurred after discharge from the OTP. Children with oedema on admission had a significantly higher case fatality rate compared to children without oedema, 6.2% (95% CI [3.5, 19.3]) versus 1.8% (95% CI [1.0–3.0]), respectively. Children with severe oedema on admission had the highest case fatality rate, 12.0% (95% CI [5.9, 20.3]). A significant number of children, that is, 6.7% (66/991), were participating in the OTP programme throughout the 14‐week follow‐up (Table 2).

At the end of 14 weeks of follow‐up, 34.6% (321/928) of children were severely malnourished, and 37.5% (348/928) were moderately malnourished; thus, 72.1% (669/928) of children admitted to the programme were acutely malnourished 14 weeks later (Table 3). Of the children with the most severe acute malnutrition on admission (MUAC < 110 mm), more than half (51.3%, 201/392) were severely malnourished 14 weeks later when using MUAC < 115 mm or oedema criteria. Proportion of children with SAM 14 weeks after initial admission was significantly higher among children with most severe degree of wasting when compared to children who were less severely wasted and oedematous on admission (Table 3).

**Table 3:** Nutritional status 14 weeks after admission to OTP by nutritional status at admission

Nutritional status at admission	Severe acute malnutrition		Moderate acute malnutrition		Normal nutritional status	
	*n* (%)	95% CI	*n* (%)	95% CI	*n* (%)	95% CI
All children (*N* = 928)	321/928 (34.6)	31.6, 37.7	348/928 (37.5)	34.4, 40.6	259 (27.9)	25.1, 30.9
All SAM (*n* = 734)	293/734 (39.9)	36.4, 43.5	272/734 (37.1)	33.6, 40.6	169 (23.0)	20.1, 26.2
Severe oedema (*n* = 64)	20/64 (31.2)	20.8, 43.3	7/64 (10.9)	4.9, 20.4	37 (57.8)	45.5, 60.4
Mild/moderate oedema (*n* = 128)	33/128 (25.8)	18.8, 33.9	39/128 (30.5)	23.0, 38.8	56 (43.8)	35.3, 52.4
MUAC < 110 mm (*n* = 392)	201/392 (51.3)	46.3, 56.2	145/392 (37.0)	32.3, 41.9	46 (11.7)	8.8, 15.2
MUAC 110–114 mm (*n* = 150)	39/150 (26.0)	19.5, 33.5	81/150 (54.0)	46.0, 61.9	30 (20.0)	14.2, 27.0
All non‐SAM (*n* = 194)	28/194 (14.4)	10.0, 19.9	76/194 (39.2)	32.5, 46.2	90 (46.4)	39.4, 53.4

*Note*. MUAC = mid‐upper arm circumference; OTP =outpatient therapeutic programme; SAM = severe acute malnutrition.

## 4 DISCUSSION

Despite adequate stay in the programme, more than one‐third of discharged children (35.8%) were severely malnourished, and 37.7% were moderately malnourished on discharge. Only 32.7% of SAM children had gained the required 15% of admission weight or had oedema resolution at discharge. Fourteen weeks after admission to the programme, 4 out of 10 children with SAM on admission remained severely malnourished and almost the same amount progressed to moderate acute malnutrition; thus, three‐quarters were acutely malnourished. Moreover, children with severe oedema on admission, that is, complicated SAM, were managed at the OTP and had a high fatality rate.

We use the same definition of recovery as programme and previous studies, that is, achieving the targeted 15% weight gain or oedema resolution at discharge (FMOH, 2007; WHO, 2013). When compared to the reported recovery rates of smaller‐scale OTPs that were not integrated into the government health system (Chaiken et al., 2006; Chamois, 2009; Collins et al., 2006; Manary, Ndkeha, Ashorn, Maleta, & Briend, 2004; Manary & Sandige, 2008), our finding of only 32.7% recovery is unacceptably low. Recovery from SAM can be delayed or hindered if the children's intake of RUTF is inadequate that could be due to limited supply by the providers (Tadesse, Ekström, & Berhane, 2016). The OTPs we studied had no external technical and financial support and were part of the government routine health services. Programmes that depend on the existing health system for provision of resources are known to suffer from shortage and stock‐out of essential programme inputs including RUTF (Puett & Guerrero, 2015). During our 6‐month stay in the field, we have noticed persistent shortage of RUTF because of lack of transportation of RUTFs from zonal/district health bureau to health posts.

Furthermore, because the actual rehabilitation of SAM takes place at the home of caregivers where chronic food insecurity often prevail and livelihood is precarious, the actual amount of RUTFs consumed by SAM children can be less than the amount provided due to sharing with other children in the household or use of RUTF as a commodity for meeting the household economic and food needs (Collins & Sadler, 2002; Tadesse, Berhane, Hjern, Olsson, & Ekstrom, 2015). Thus, an improvement in the nutritional status of children admitted to the OTP greatly depends on the household contexts and caregivers' perceptions and abilities of usage of RUTFs (Tadesse et al., 2015).

The use of percentage gain as the discharge criterion is suggested to “disfavour” the recovery of children with the lowest MUAC (Dale, Myatt, Prudhon, & Briend, 2013; Forsen, Tadesse, Berhane, & Ekstrom, 2013) and may contribute to the high proportion of children still suffering from SAM at discharge and the high number of readmissions in our study. More than half of the children with the most SAM on admission (MUAC < 110 mm) still had SAM 14 weeks later. A recent WHO update on the management of SAM recommends a shift to MUAC‐based discharge criteria instead of 15% weight gain, which is believed to increase the duration of admission to OTP, thus, allow adequate treatment (WHO, 2013). However, we noted that the majority of SAM children who stayed longer than the WHO‐recommended duration still remained malnourished, suggesting that a longer duration in OTP may not be sufficient to solve the problem. Further, during data collection, it was noticed that health extension workers appeared to discharge children based on length of stay in the OTP rather than the percentage weight gain, possibly because of instructions on a maximum duration of stay in OTP.

The occurrence of oedema was high in the children we studied when compared to another study conducted in Northern Ethiopia (Yebyo, Kendall, Nigusse, & Lemma, 2013). Children with severe oedema are considered as complicated SAM by WHO as well as by national guidelines, and, thus, they should have been referred to inpatient care for appropriate management instead of being managed in the OTPs (FMOH, 2007; WHO et al., 2007). The lack of referral might be a result of an inability of health extension workers to identify these children as having complicated SAM, as argued in another study from Ethiopia (Yebyo et al., 2013). Further, caregivers may have refused to have their children referred because of perceived difficulties in managing their ordinary household chores, including the care of other children while having children admitted to inpatient facilities (Ashworth, 2006; Ciliberto, Manary, Ndekha, Briend, & Ashorn, 2006). In addition, health extension workers may have perceived that inpatient care was not available or that caregivers would not attend if referred.

Children with severe oedema on admission had an increased risk of remaining severely malnourished 14 weeks later and also had a high risk of dying. The dietary management with RUTF in an OTP is not suitable for children with severe oedema as it contains higher protein and energy than the recommended F‐75 milk‐based diet (Collins & Sadler, 2002). Collins et al. (2006) attributed the death of oedematous children in their study to an inappropriate use of RUTF in children with oedema (Briend, 2014; Sadler, Myatt, Feleke, & Collins, 2007). This management may also have contributed to the high mortality of this group in our study. Although inpatient management would have been appropriate for these children, data on the performance of inpatient care for the management of severe oedema and other complicated cases of SAM in the study area are not available. Thus, it is not known whether these complicated SAM cases would have had a different (better) outcome in inpatient care.

Most studies assessing SAM management in OTP are based on data excerpted from programme records and performed by organizations involved in the programme implementation. To maximize the quality of collected data, our study based the results on an independent research team that collected data and assessed the children's nutritional status. The research team was not involved in any aspect of programme service provision or other health and nutrition interventions in the study area. All data collectors went through careful training in anthropometric measurements including standardization sessions prior to and throughout the data collection period. A potential limitation of this study was the inclusion of all discharged children in the analysis of programme outcome regardless of longer duration between discharge date and our nutritional assessment after discharge of children from OTP. However, because there was no significant difference in proportion of children with SAM according to duration since discharge, our finding of low recovery (achieving programme criteria for recovery) is likely to be a valid estimate of achievement of programme recovery criteria.

## 5 CONCLUSION

Despite children participating in the OTPs for the recommended duration of the programme, many were discharged without reaching the required weight gain and still acutely malnourished. Children with severe oedema on admission had a higher fatality rate than those with nonoedematous SAM. Further research is needed to understand the contextual factors that constrain the management of SAM at service points and at home.

## CONFLICTS OF INTEREST

The authors declare that they have no conflicts of interest.

## CONTRIBUTIONS

EE and YB conceived and designed the study. ET and AW performed the field work, and EE and YB provided supervision throughout the research and writing process. ET carried the analysed data and drafted the paper in close collaboration with EE. All authors revised and approved the final version of the paper.
